# An Exploration Based Cognitive Bias Test for Mice: Effects of Handling Method and Stereotypic Behaviour

**DOI:** 10.1371/journal.pone.0130718

**Published:** 2015-07-08

**Authors:** Janja Novak, Jeremy D. Bailoo, Luca Melotti, Jonas Rommen, Hanno Würbel

**Affiliations:** 1 Division of Animal Welfare, VPH Institute, University of Bern, Länggassstrasse 120, 3012, Bern, Switzerland; 2 Department of Zoological Systematics and Evolution, Faculty of Biology, University of Marburg, Biegenstraße 10, 35037, Marburg, Germany; University of Houston, UNITED STATES

## Abstract

Behavioural tests to assess affective states are widely used in human research and have recently been extended to animals. These tests assume that affective state influences cognitive processing, and that animals in a negative affective state interpret ambiguous information as expecting a negative outcome (displaying a negative cognitive bias). Most of these tests however, require long discrimination training. The aim of the study was to validate an exploration based cognitive bias test, using two different handling methods, as previous studies have shown that standard tail handling of mice increases physiological and behavioural measures of anxiety compared to cupped handling. Therefore, we hypothesised that tail handled mice would display a negative cognitive bias. We handled 28 female CD-1 mice for 16 weeks using either tail handling or cupped handling. The mice were then trained in an eight arm radial maze, where two adjacent arms predicted a positive outcome (darkness and food), while the two opposite arms predicted a negative outcome (no food, white noise and light). After six days of training, the mice were also given access to the four previously unavailable intermediate ambiguous arms of the radial maze and tested for cognitive bias. We were unable to validate this test, as mice from both handling groups displayed a similar pattern of exploration. Furthermore, we examined whether maze exploration is affected by the expression of stereotypic behaviour in the home cage. Mice with higher levels of stereotypic behaviour spent more time in positive arms and avoided ambiguous arms, displaying a negative cognitive bias. While this test needs further validation, our results indicate that it may allow the assessment of affective state in mice with minimal training—a major confound in current cognitive bias paradigms.

## Introduction

Assessment of the subjective component of animal welfare has mostly relied on physiological [1,2] and behavioural [3–5] measures. While these measures are generally good indicators of arousal (i.e. intensity), they do not necessarily provide unambiguous information about the valence of an affective state (i.e. positive or negative) [6]. Instead, valence of affective state appears to be more closely related to changes in cognitive processing [7]. Thus, based on evidence that cognitive processes such as attention, judgement and memory are biased by the valence of an individual’s affective state [7–9], measures of cognitive bias have recently been implemented as proxy measures of affective valence in the study of animal welfare [6,10].

Among tests probing such cognitive biases, the most commonly used type is a decision making paradigm which uses judgement biases to infer animals’ affective state. In a judgement bias test, an animal is typically trained to discriminate between two different stimuli, one predicting a positive outcome and the other a less positive or negative one. Once the discrimination is learned, the animal is presented with one or more ambiguous stimuli to assess whether it expects a positive outcome, suggesting the animal is in a positive affective state, or whether it expects a negative outcome, suggesting it is in a negative affective state [6].

While many cognitive bias tests exist for rats [11–16], only a few have been implemented in mice [17,18]; the most widely used laboratory animal [19]. One problem with these tests is the length of training required for the animals to learn to discriminate between the positive and negative stimuli [6]. Furthermore, prolonged daily handling involved in training and testing may influence their affective state, thereby confounding the results [20,21]. To reduce training time, one test variant used approach latency in a spatial go/no-go paradigm, using location as a stimulus [12,14,16,18]. This paradigm is easier to learn because of the strong salience of spatial location and its relevance to foraging behaviour in rodents [22–24]. For example, using a spatial cognitive bias test, Burman et al. [16] found that rats housed in standard cages have longer approach latencies to intermediate (ambiguous) locations compared to enriched housed rats, which was interpreted as a negative cognitive bias. Similarly, negative cognitive biases were found in rats exposed to bright light [12], congenitally helpless rats (a genetic model of depression) [14] and 5-HTT knock-out mice [18], which were more reluctant to approach ambiguous locations compared to control animals. However, approach latencies can be confounded by motivational factors associated with affective states [25], making interpretation difficult. Other paradigms are therefore needed to reduce training time and avoid adverse handling effects.

Tests based on free exploration may be promising in this respect as there is some evidence that changes in exploratory behaviour are associated with variation in affective state. For example, in a spatial discrimination task [26], removal of environmental enrichment (a negative event) has been shown to reduce the extent to which rats explored previously inaccessible (i.e., ambiguous) arms of a radial arm maze. Rats showed a higher preference for familiar arms, indicating that reduced welfare (induced by removal of enrichment) may override the animal’s natural tendency to explore ambiguous, potentially aversive environments [27]. The present study expanded upon this idea and used a “free choice” spatial discrimination test on an eight-arm radial maze to assess affective state in mice. Similar to above mentioned spatial cognitive bias tests, the mice were trained to associate the entry of two adjacent arms with a positive outcome (positive arms; darkness and food), while entering the two opposite arms predicted a negative outcome (negative arms; light on and white noise). After six days of training, mice were tested for affective valence by providing them with access also to the intermediate (ambiguous) arms that were previously unavailable for exploration. However, instead of using latency to approach to measure cognitive bias, mice were left to explore the maze, exploiting the conflict between the exploratory drive and neophobic tendency when faced with novel or ambiguous places [28–30].

To validate the test, we used two different handling methods to manipulate the animals’ affective state. While handling has been shown to induce an acute stress response in mice [2], repeated handling can attenuate aversion toward human contact [31,32]. It has been argued that such habituation may ensure that the animals’ responses in experiments are task specific [33–35]. However, recent studies suggest that the type of handling can influence both physiological and behavioural measures of stress differently [20,21]. More specifically, the standard method of picking mice up by their tails has been shown to induce greater anxiety compared to more gentle methods (cupping and tunnel handling;[20,21]). If tail handling is anxiogenic, regular handling by this method may result in chronic stress and anxiety, thereby compromising the welfare of the animals (but see [36,37]). By altering the behaviour and physiology of mice, handling may also be an important source of uncontrolled variation in many animal experiments [37,38]. The primary aim of the study was therefore to use two different handling methods (as used by Hurst and West [20]) to validate an exploration based cognitive bias test. Consistent with the cognitive bias theory [6,39], we hypothesized that tail handled mice (being in a more negative affective state) would show reduced exploration of ambiguous arms compared to cupped mice. Specifically, tail handled mice were expected to spend less time in the near negative ambiguous arms, indicating a more pessimistic exploration style based on a higher expectation of negative events.

Conditions inducing anxiety and stress during the pre and post weaning phases are also associated with the development of abnormal repetitive behaviour, such as stereotypies [40–43]. For example, the level of stereotypic behaviour in adult laboratory mice was related to their corticosterone response to weaning [44,45]. Furthermore, some stereotypies in laboratory rodents may develop from a motivation to escape [46,47], as a response to missing resources (e.g., the absence of burrows [48]) or after removal of environmental enrichment [41]. This indicates that frustration or behavioural thwarting may be associated with the development of stereotypies. Attempts to link stereotypic behaviour with poor welfare, however, have yielded mixed results (for a review see [49]), and non-stereotypic animals, for example, have been shown to exhibit a stronger physiological response to stressors [50,51].

Therefore, a secondary aim of this study involved exploration of whether and how stereotypy performance affects cognitive bias. If stereotypy performance in the home cage is indicative of negative affective states in mice, higher levels of stereotypy would be expected to result in reduced exploration of the ambiguous arms of the maze, especially the near negative ambiguous arms.

## Materials and Methods

### Animals and husbandry

28 female CD-1 outbred mice (Harlan laboratories, Netherlands) from four litters (n = 7 per litter) were obtained at three weeks of age and allocated in pairs to cages so that each cage contained mice from two different litters. The mice were housed in Type II cages, with two Kleenex tissues as nesting material provided weekly. Food and water were provided *ad libitum*. Subjects were kept on a 12:12 hour dark:light cycle, with lights off at 9 AM.

### Experimental design

**Fig 1:**
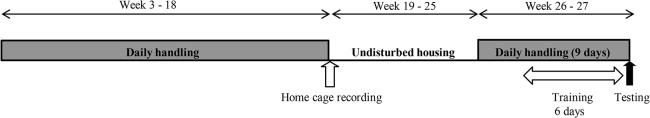
Timeline of the study. The initial handling phase (week 3–18) was part of a study reported elsewhere [52].

From the time of arrival, for the duration of 15 weeks, the mice were handled daily by one experimenter (J.R.) using two different handling methods (tail handling, cupped handling) to assess the effect of handling method on stereotypy development [52]. One mouse died during this period so that cage was excluded from the present study. Data on stereotypic behaviour were obtained from video recordings of home cage behaviour performed at the end of the handling phase at 18 weeks of age. From week 19 to 25, the mice remained undisturbed, with weekly cage changes and health checks using the same handling methods previously assigned (i.e. tail handling or cupped handling). Starting at 26 weeks of age, the mice were again handled daily by another experimenter (J.N.) for nine days using the same handling method as before. After three days into the handling phase, spatial discrimination training on an eight arm radial maze was initiated. The mice were trained once daily for six consecutive days before being tested. Throughout training the mice continued to be handled daily while no handling occurred on the day of testing (Fig 1).

### Home cage behaviour

Home cage behaviour was recorded at 18 weeks of age using infrared sensitive cameras (VC Video components GmbH, Germany). Behaviour was recorded during the dark phase for two consecutive days when the mice were not handled. For individual recognition, one mouse per cage was marked individually using a black permanent marker (Edding 500) while the cage mate was sham-marked. Marking occurred one day before home cage recording. Observations were restricted to the 2^nd^, 3^rd^, 6^th^ and 7^th^ hour of the dark phase, as pilot observations indicated activity peaks during these hours. Each mouse was observed for the first 5 min of every 20 min interval, and behaviour was sampled using one-zero sampling with 30 s intervals [53], yielding 30 observations per mouse per hour, and 240 observations per mouse in total. General activity and stereotypic behaviour were recorded (see Table 1). To avoid observer bias, cages were relabelled by another person before recordings, and video recordings were scored blind to the treatment condition.

**Table 1:** Ethogram for home cage behaviour recording.

Category	Name	Definition
**General activity**	Inactive	Sitting or lying motionless throughout the 30 s interval, occasionally interrupted by brief single twitches lasting no longer than 5 s.
	Active	All activities except the stereotypic activities listed below.
**Stereotypic behaviour**	Bar-mouthing	Chewing on a bar with the bar held in the gap between incisors and molars (diastema) while hanging on the cage lid (with all four paws or the forepaws only) or standing on the hind legs. Bar-mouthing may be performed on the spot or by moving along the bar while chewing.
	Circling	Running around the cage in circles.
	Cage-top twirling	Spinning around the longitudinal body axis while hanging on the cage lid with the forepaws.
	Back-flipping	Backward flip from one cage wall or the food rack towards the opposite cage wall, with or without touching the cage lid and/or the opposite cage wall during the flip.

Behaviour patterns were considered as stereotypic if the same movement sequence was repeated continuously for at least 10 s without pauses longer than 3 s (bar-mouthing) or at least three times in a row without pauses longer than 3 s between bouts (cage-top twirling, back-flipping).

### Experimenter handling

Handling occurred from 10–11 AM according to the method described by Hurst and West [20]. Briefly, the two mice in each cage were randomly assigned to either the tail handling or cupped handling treatment. Handling order within cages was alternated daily so that each mouse got handled first on alternate days. For tail handling, mice were grasped by the base of their tail with thumb and forefinger of one hand and lifted onto the palm of the other hand where they were held by the tail for 30 s before being returned to the cage. For cupped handling, mice were lifted up on one or both open hands and left to sit on the hand or move around unrestrained for 30 s before being returned to the cage.

### Radial maze cognitive bias test

#### Apparatus

**Fig 2:**
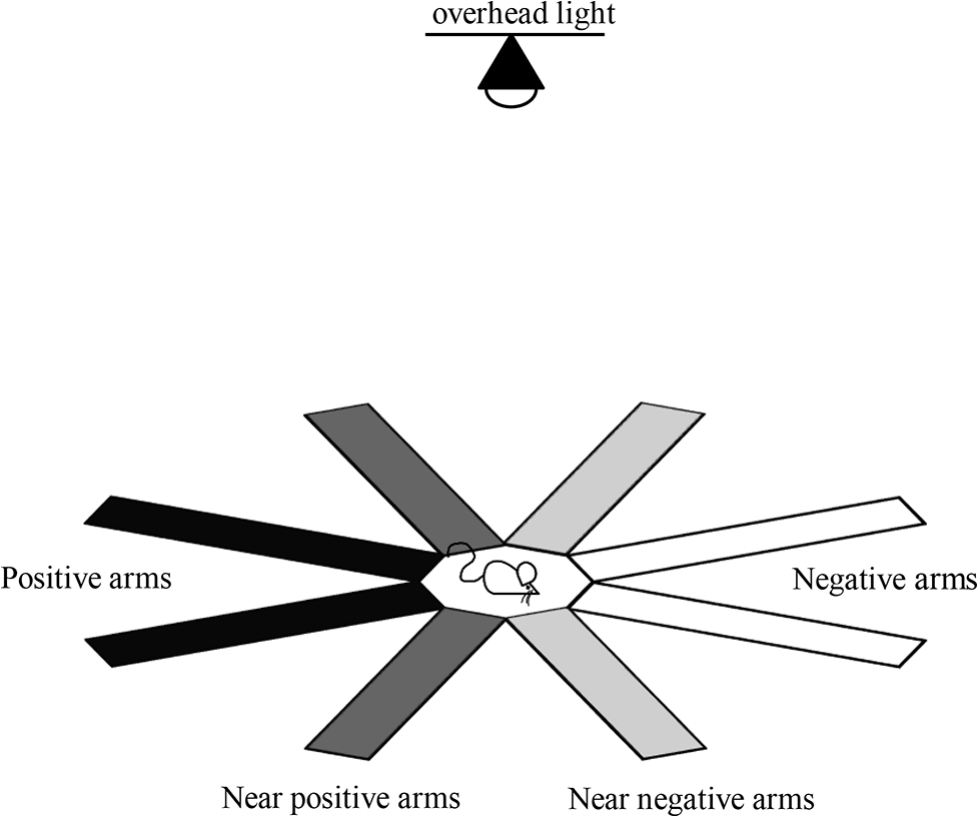
Radial arm maze. Two positive (black) and two negative reference arms (white) and two near positive (dark grey) and two near negative ambiguous arms (light grey).

Mice were trained and tested on an eight arm radial maze (Med-Associates Inc.; Fig 2). Each arm was 46 cm long and 9 cm wide and the diameter of the central arena was 28 cm. The bottom of the maze was backlit with infrared light which eliminated tracking errors associated with automated tracking [54]. A computer with Ethovision XT software (Noldus, Version 9) recorded the animal’s movement in the maze via a video camera equipped with an infrared pass filter and automatically activated contingencies when the animal entered an arm or the end of an arm. The detection settings for EthoVision XT were selected so that both the percentage of samples in which the subject was not found and the percentage of samples skipped were less than 1% per trial.

#### Training and testing

Training started at 12 PM, one hour after the last cage was handled. For training, the two positive and two negative reference arms were open and the ambiguous arms closed. Mice were trained for one ten minute session each day, for six consecutive days. The order of training of the two mice from a cage was the same as the order of handling for each respective day.

Each session started with the overhead light on (400 lux). Reaching the ends of either of the positive arms, turned the overhead light off either until the mouse entered a negative arm or until it exited a positive arm and stayed in the central arena for 20s. Reaching the end of the positive arms also activated a pellet dispenser, dispensing a 20 mg chocolate flavoured pellet (Dustless Precision Pellets, Bio-Serv). Pellet dispensers were regulated automatically, but there were two instances where the dispenser malfunctioned and no pellets were dispensed. For these instances the pellet was dispensed manually. When entering either of the two negative arms, the overhead light came on and stayed on until the animal entered the end of a positive arm. Entering the end of negative arms also triggered a burst of white noise which remained on until the animal exited the negative arm.

After six days of training, the mice were tested for responses to ambiguous arms during one ten minute test session in which all eight arms of the maze were open. The test session started with the overhead light on, and the activated contingencies for the positive and negative arms remained the same as in the training phase, whereas entering ambiguous arms and reaching the ends of ambiguous arms activated no contingencies.

For both training and testing, the time spent in each arm, the number of arm entries, as well as the running speed was recorded for each mouse. One mouse that performed circling behaviour in the central arena of the radial maze was excluded from the analysis as it never performed the test.

### Ethical Statement

This study was carried out in strict accordance with the recommendations in the Animal Welfare Ordinance (TSchV 455.1) of the Swiss Federal Food Safety and Veterinary Office. It was approved by the Cantonal Veterinary Office in Bern, Switzerland (Permit Number: BE12/12).

### Statistical Analyses

All statistical analyses were performed with R (version 2.15.3) and R Studio (version 0.98.507). P-values below 0.05 were considered significant for all analyses and the function lmer in the R package “lmer4” and “lmerTest” [55] was used to fit linear mixed effects models. The assumptions of normally distributed errors and homogeneity of variance were examined graphically with the use of the Normal plot and the Tukey-Anscombe plot. To satisfy these assumptions, level of stereotypic behaviour was square-root transformed. Handling method was included in the model as a fixed effect, litter and cage as random effects and stereotypy level as a covariate. Results shown are untransformed means ± SEM. Time spent in arms is presented relative to trial duration. For the primary models run, cage and litter had no effect on any of the outcome measures, so they were excluded from subsequent *post hoc* analyses. Time spent in arms was taken as a primary measure of arm preference. Since time spent in different arms during a single session was not independent, we calculated a “reference arm score”, subtracting time spent in all ambiguous arms from time spent in all reference arms, divided by the time spent in all arms, and used the score as an outcome variable in the model. A higher score indicated that mice spent more time in reference arms and less time in ambiguous arms. Similarly, a “positive arm score” was calculated as time spent in negative arms subtracted from time spent in positive arms, divided by time spent in all reference arms. An “near positive arm score” was calculated for time spent in the four ambiguous arms by subtracting time spent in near negative arms from time spent in near positive arms, dividing it by the time spent in all ambiguous arms. Number of arm entries was used as a secondary measure of arm preference and is presented relative to all arms entered. These outcome measures were then used to assess effects of handling method and stereotypy level on exploration of the maze. Additionally, to assess activity in the radial maze, we recorded the total number of arms entered and running speed. For test data and for comparison of the results of the last training session and the test phase, preferences among the arms were evaluated *post hoc* with Bonferroni corrected pair sampled t-tests.

## Results

### Effect of handling method on cognitive bias

Experimenter handling did not affect any of the measures of maze exploration during training or testing. A summary of these analyses is presented in Table 2.

**Table 2:** Results of experimenter handling on exploratory measures in the eight arm radial maze.

Training	*Tail handling*	*Cupped handling*	*F_1_*,*_21_ (Handling)*	*F_1_*,*_105_ (Handling x session)*
positive arm score	0.18 ± 0.03	0.13 ± 0.05	0.31	0.28
number of arms entered	43 ± 2	49 ± 1	4.50	0.57
positive arms entered %	58 ± 2	58 ± 1	0.02	0.67
negative arms entered %	42 ± 1	42 ± 1	0.02	0.67
**Testing**				
reference arm score	- 0.13 ± 0.05	- 0.25 ± 0.07	1.43	-
positive arm score	0.17 ± 0.10	0.36 ± 0.02	3.16	-
near positive arm score	0.13 ± 0.07	0.12 ± 0.05	0.28	-
reference arms entered	0.44 ± 0.01	0.39 ± 0.03	2.67	-
ambiguous arms entered	0.56 ± 0.01	0.61 ± 0.03	2.67	-
number of all arms entered	52 ± 5	60 ± 4	1.79	-
speed (cm/s)	10 ± 1	11 ± 1	0.68	-

For both handling methods, mean ± SEM are shown. Time spent in arms is presented as proportion of trial duration. Higher positive arm score indicates mice spent more time in positive arms compared to negative arms, higher reference arm score indicates mice spent more time in reference arms compared to ambiguous arms, and a higher near positive arm score means more time spent in near positive versus near negative arms. Number of all arms entered is presented as counts and arm entries to reference and ambiguous as proportion of all arms entered.

### Radial maze cognitive bias test

#### Training

**Fig 3:**
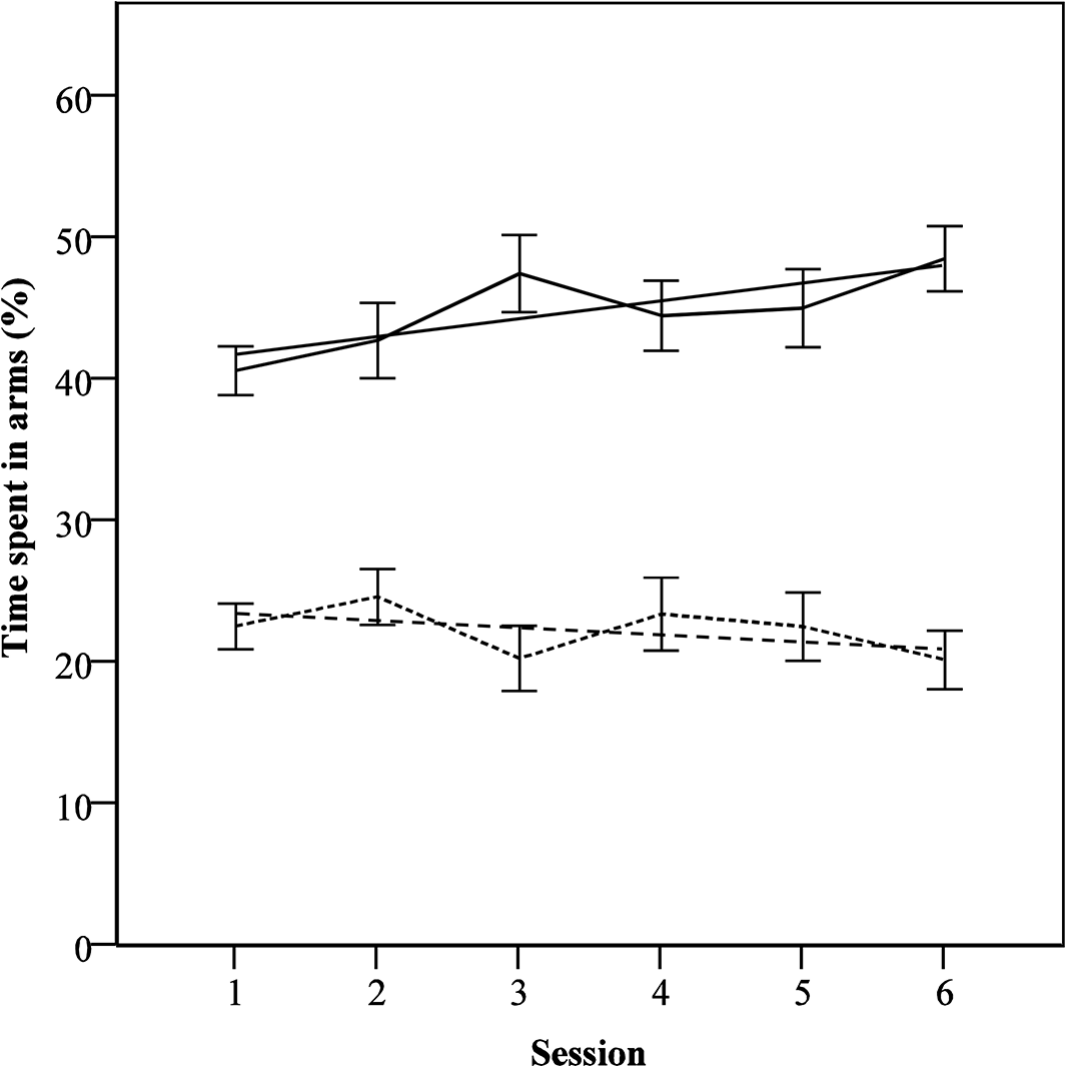
Time spent in positive and negative arms across training sessions with added regression lines. Time spent in positive and negative arms are inversely related and both are shown for reference purposes only. Time spent in arms is shown as proportion of trial duration, mean ± SEM.

Mice spent progressively more time in positive arms across training sessions (*F*_5,115_ = 3.64, *p* < 0.05, Fig 3), and correspondingly, spent less time in the negative arms, although the time spent in negative arms did not change significantly during training (*F*_5,115_ = 1.77, *p* > 0.05).

Moreover, while the number of entries to positive arms increased across training sessions (*F*_5,115_ = 5.15, *p* < 0.05), the number of entries to negative arms decreased (*F*_5,115_ = 20.26, *p* < 0.05), indicating increasing discrimination.

Furthermore, general activity on the maze decreased across training sessions as indicated by a reduction in the total number of arms entered (*F*_5,115_ = 7.20, *p* < 0.05) from 53 ± 3 arms entered on day one, to 42 ± 2 on day six. Reduced activity was also evidenced by a decrease in running speed (*F*_5,115_ = 6.74, *p* < 0.05) across sessions, from 9 ± 0.44 cm/s to 8 ± 0.37 cm/s.

#### Testing

**Fig 4:**
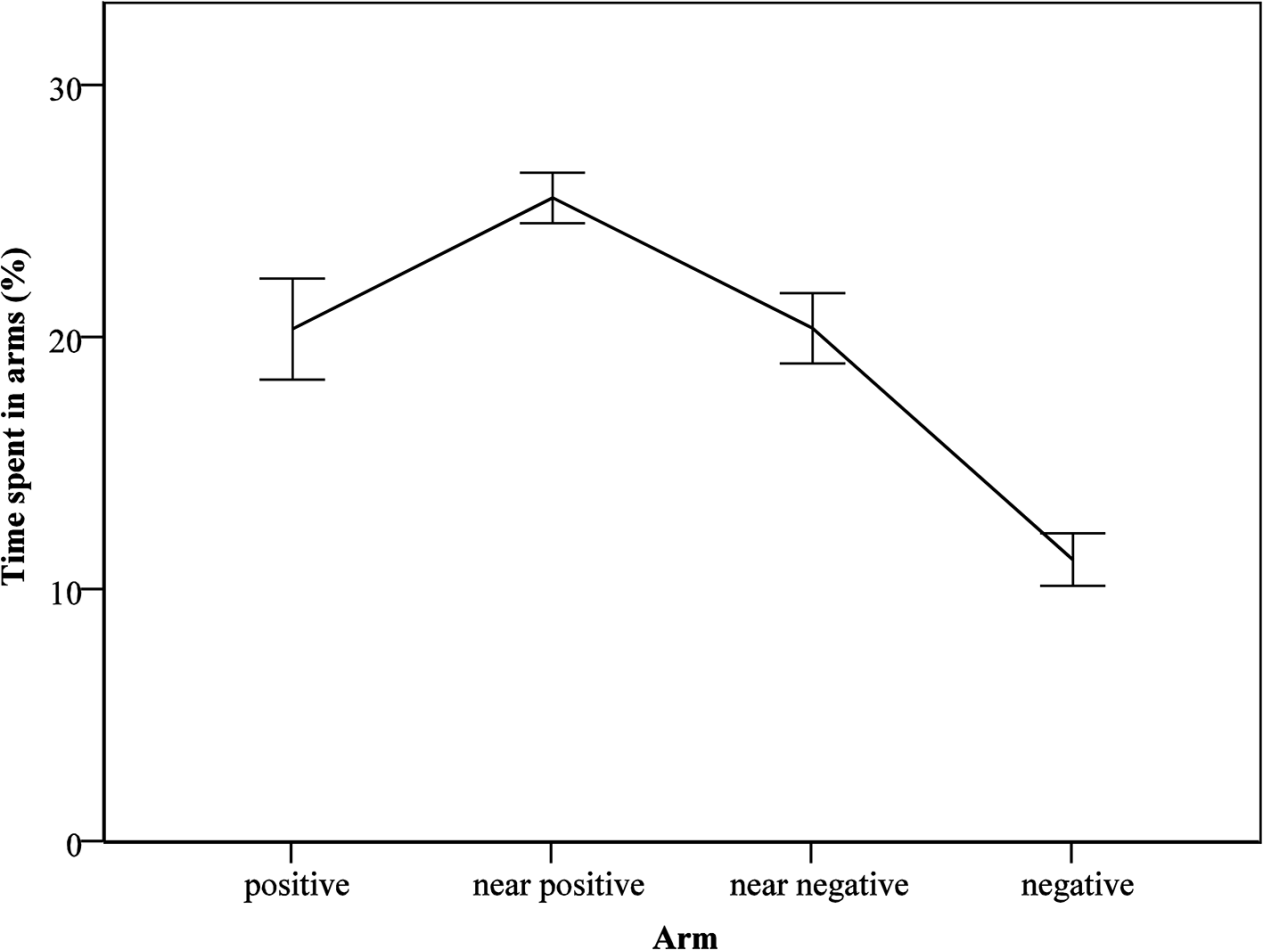
Time spent in each type of arm during the test session. Time spent in arms is shown as proportion of trial duration, mean ± SEM.

As during training, the mice showed a preference for positive arms over negative arms (*t*(24) = 4.83, *p* < 0.05), spending 20% ± 2% of the time in the positive arms as opposed to 11% ± 1% in the negative arms. However, the time spent in positive arms was reduced compared to the last training session (*t*(24) = 2.53, *p* < 0.05), from 49% ± 2% to 20% ± 2%, because during testing twice as many arms were available to explore. Overall, the mice spent more time in ambiguous arms (46% ± 2%) than in reference arms (31% ± 2%; *t*(24) = 4.42, *p* < 0.05). Moreover, they discriminated between the ambiguous arms, spending more time in the near positive arms (26% ± 1%) than on the near negative arms (20% ± 2%; *t*(24) = 2.94, *p* < 0.05; Fig 4).

Mice were more active during the test session compared to the last training session, as they entered more arms (*t*(24) = 6.90, *p* < 0.05) and moved faster (*t*(24) = 4.99, *p* < 0.05).

### The relation between stereotypy performance and cognitive bias

#### Home cage behaviour and stereotypy performance

**Fig 5:**
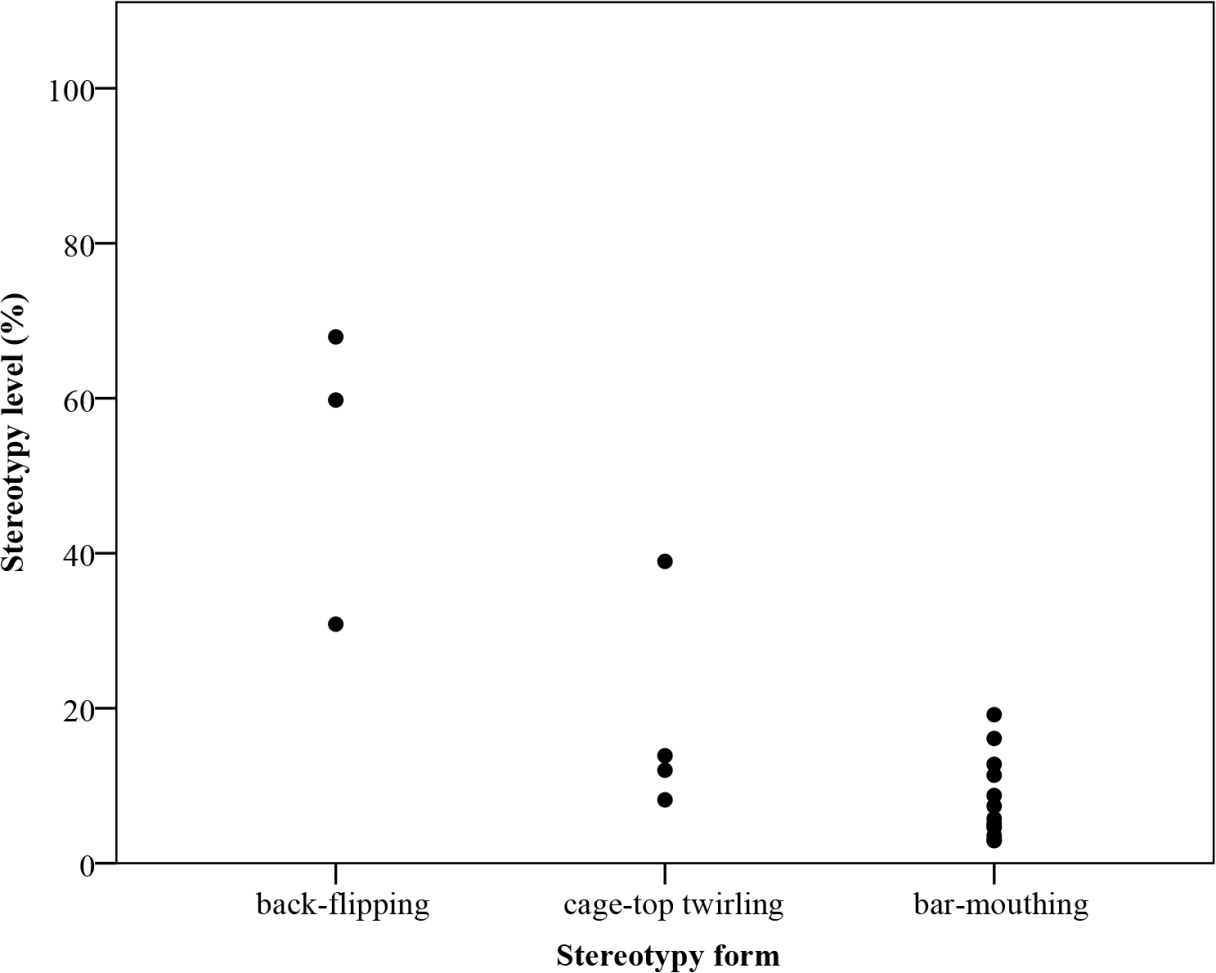
Level of different forms of stereotypic behaviour. Bar-mouthing (n = 13), cage-top twirling (n = 4), back-flipping (n = 3).

Mice were active during 73% ± 3% of the observed time and spent 13% ± 3% of their active time performing stereotypic behaviour. They performed three forms of stereotypic behaviour; bar-mouthing (n = 13), cage-top twirling (n = 4) and back-flipping (n = 3), while six mice performed no stereotypic behaviour. The relationship between the incidence of the different forms of stereotypy and the level of expression is presented in Fig 5. These data show that mice performing back-flipping did so at a higher level compared to mice performing bar-mouthing or cage-top twirling (Kruskall-Wallis; χ^2^(2) = 9.35, *p* < 0.05), indicating that the level of stereotypy performance was partly confounded by the form of stereotypy.

**Fig 6:**
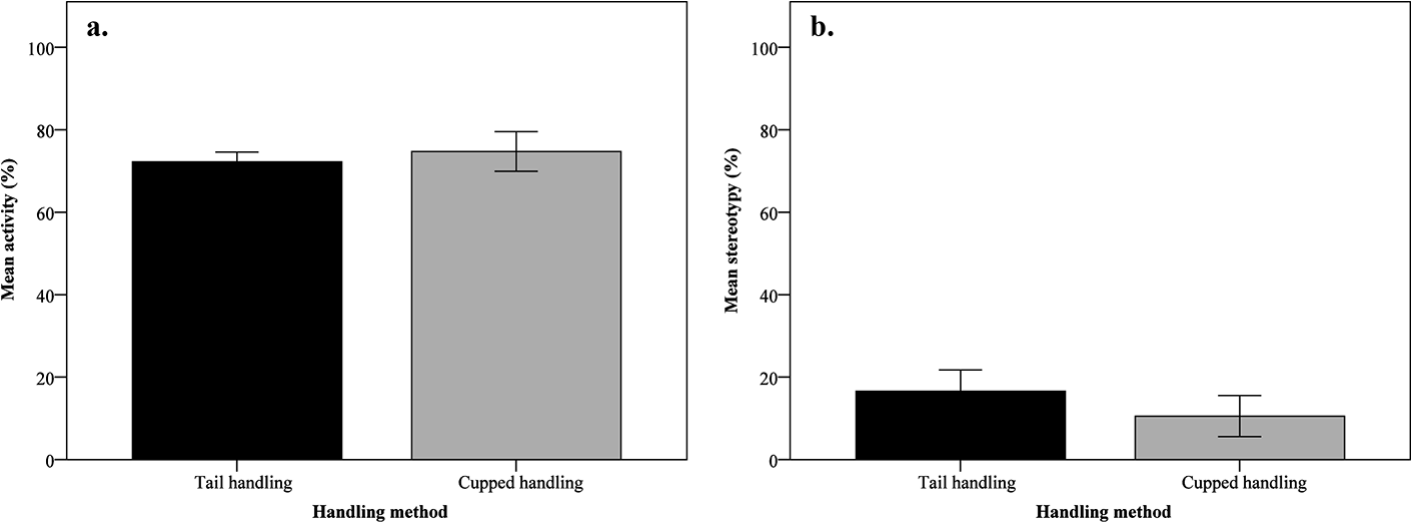
Level of (a.) home cage activity and (b.) stereotypic behaviour in both handling groups. Home cage activity is presented as proportion of observed time and stereotypic behaviour as proportion of active time, mean ± SEM.

Handling method had no effect on home cage activity (*F*_1,24_ = 0.20, *p* > 0.05) and stereotypy level (*F*_1,24_ = 1.39, *p* > 0.05; Fig 6).

#### Training

The level of stereotypic behaviour affected time spent in different arms during training. Mice with higher level of stereotypy spent more time in positive arms (positive arm score; *F*_1,115_ = 5.33, *p* < 0.05). They were also more active; entering more arms (*F*_1,115_ = 34.32, *p* < 0.05) and running faster (*F*_1,115_ = 6.74, *p* < 0.05).

#### Testing

Exploration of the different arms was affected by stereotypy level. Consistent with the training data, mice with a higher level of stereotypic behaviour spent more time in positive arms during testing (positive arm score; *F*_1,24_ = 14.53, *p* < 0.05), despite no effect on the number of positive arms entered (*F*_1,24_ = 2.61, *p* > 0.05). They also spent more time in reference arms and avoided ambiguous arms (reference arm score; *F*_1,24_ = 4.97, *p* < 0.05). More specifically, they showed greater avoidance of near negative arms (near positive arm score; *F*_1,24_ = 31.66, *p* < 0.05). Stereotypy level did not predict number of chocolate pellets eaten during the test phase (*F*_1,24_ = 0.66, *p* > 0.05). It also did not affect activity during the test session as measured by the number of arms entered (*F*_1,24_ = 3.29, *p* > 0.05) and speed (*F*_1,24_ = 4.08, *p* > 0.05).

The relationship between the different forms of stereotypic behaviour and maze performance was not analysed because of the small sample size per stereotypy form.

## Discussion

We used different handling methods to assess the validity of a new exploration based paradigm for assessing affective states in mice. Mice were trained on a radial arm maze to discriminate between positively cued arms (positive arms) and negatively cued arms (negative arms) and were tested for exploration of positive and negative arms as well as intermediate ambiguous arms that had not been accessible during training. We found no effect of handling method on radial maze exploration. Furthermore, we examined if mice with higher stereotypy levels displayed reduced exploration of ambiguous arms. Mice with higher stereotypy levels spent more time in positive arms during both training and test phase and avoided near negative ambiguous arms. However, mice performing back-flipping displayed higher levels of stereotypic behaviour than mice performing other forms of stereotypy, indicating that the relationship between the level of stereotypy and exploration of the radial maze was confounded by the form of stereotypy performed.

### Effects of handling method on cognitive bias

The two different handling methods did not affect exploration of the radial maze, thus we failed to formally validate the test. Mice of both handling groups spent more time in ambiguous arms compared to reference arms, indicating a strong motivation to explore novel, ambiguous spaces overriding their neophobic tendency [26,56,57]. There are several possible explanations for the lack of handling effect. First, the test might not be appropriate or sensitive enough to detect changes in affective state. However, mice discriminated between the two reference and the two ambiguous arms, indicating different expectations of positive and negative outcomes. Another possible explanation for the lack of treatment effect is that the test did not detect differences in affective states induced by handling. While Franks et al. [26] found that removal of environmental enrichment induced a reduced exploration of ambiguous arms in rats, the effects of handling may have been too subtle to induce changes in maze performance. Different treatments known to induce measureable changes in interpretation of ambiguous stimuli (e.g. removal of enrichment [26] or pharmacological manipulation [58,59]) should therefore be used in future studies to assess the validity of this test for assessing variation in affective state in mice. Additionally, the lack of a handling effect may reflect the mixed effects of handling on other measures of affective state in previous studies. While Hurst and colleagues [20,21] report an anxiogenic effect of tail handling in CD-1, BALB/c and C57BL/6 mice, others have shown that mice may adapt to experimenter handling [36,37]. However, the present study also differed from that by Hurst and West [20] in that mice were handled daily for many weeks as opposed to only nine days. Thus, the mice in the present study may have habituated to repeated daily handling.

Despite the absence of handling effects, however, our data suggest that this test has potential for measuring affective states in mice but requires further validation. Discrimination of positive and negative arms was evident from the first training session onwards, which renders the test quick to implement and indicates that the number of training sessions may be reduced even further. Many tests designed to measure affective state have problems with long and frequent training sessions [6,15,60,61], and a test that does not require extensive training of animals may reduce experimenter error and bias considerably. Furthermore, the mice clearly discriminated between the two ambiguous arms as they spent more time in the near positive and less time in the near negative arms. This parallels results of conventional spatial cognitive bias tests [12,14,16], suggesting that the mice associated near negative arms more strongly with a negative outcome than near positive arms. Analysis of exploration of the two types of ambiguous arms may allow the detection of specific biases at either or both ambiguous locations, which could help to discriminate between affective states of the same valence [6,10]. For example, it has been hypothesised that avoidance of near negative ambiguous cues is indicative of anxiety, while avoidance of near positive cues is linked to anhedonia characteristic of depressive-like states [6,12].

While this test uses spatial discrimination, similar to other spatial cognitive bias tests [12,16], it differs in the outcome measure used. Other spatial tests use approach latency while the present test is a preference based test, where the animal is free to explore all arms of the maze. Therefore, the difference in exploration of near positive versus near negative arms could simply reflect the preference of positive over negative arms increasing the likelihood of entering near positive as opposed to near negative arms. However, during the test session, mice spent almost a fifth of the trial time in the central arena, suggesting they did not simply transverse from one arm to another but took time before entering an arm. Thus, it is unlikely that the preference for near positive arms was influenced by their proximity to positive arms.

### The relation between stereotypy performance and cognitive bias

Stereotypy level affected exploration of the maze during both training and test sessions. During training, mice with higher levels of stereotypy visited more arms overall and moved faster. This is consistent with high stereotyping animals generally being more active [22,42,62], although higher activity was only observed during the training phase.

Stereotypy performance did not affect discrimination between positive and negative arms, which is consistent with other studies showing that stereotypies do not impair simple learning tests [22]. However, mice with higher levels of stereotypy spent more time in positive arms during training and testing, and spent more time in the reference arms compared to the ambiguous arms, indicating a reluctance to forgo known environments to explore ambiguous arms. In a similar radial maze paradigm, rats which had experienced removal of enrichment showed lower levels of exploration of ambiguous environments, by spending more time in positive arms [26]. Furthermore, reduced exploration of ambiguous arms was not a result of reduced activity as overall activity in the maze was similar for all mice. A possible explanation for increased preference for positive arms in high stereotyping mice may have been that higher stereotypy (and activity) levels may cause higher energy demands. However, we found no evidence for high stereotypy mice consuming more chocolate pellets.

Cage induced stereotypies have been shown to reflect an inability to inhibit motor responses [22], and a possible explanation for high stereotyping mice spending more time in reference arms and avoiding ambiguous arms is that they simply failed to inhibit entering the familiar arms. Evidence from pharmacological and behavioural studies suggests that cage induced stereotypies are caused by a disinhibition of behavioural control mechanisms [63,64]. Disruption of activation and inhibition of corticostriatal circuits, which connect the basal ganglia with the cerebral cortex can result in dysfunctions related to the initiation, inhibition and control of movement [65–67]. Indeed, animals with disrupted cortical basal ganglia pathways display perseverative behaviour, which is defined as a tendency to repeat a previously learned behavioural response [22,68].

While we cannot entirely exclude behavioural inflexibility as a possible explanation for high stereotyping mice spending more time in reference arms, this would not explain why mice preferred near positive to near negative ambiguous arms. Furthermore, stereotypy level only affected time spent in near negative arms but not time spent on near positive arms. This indicates that high stereotyping mice selectively distinguished between ambiguous arms and perceived near negative arms more negatively. It further suggests that this difference does not simply reflect the preference of positive over negative arms increasing the likelihood of entering near positive arms as discussed above. This is consistent with other spatial cognitive bias studies [12,14,16,18] and may indicate an increased expectation of aversive events in near negative but not in near positive arms, thus a negative cognitive bias [16,61]. Additionally, while high stereotypy levels are linked to perseverative responses in some strains of mice [69], this could not be replicated in the CD-1 strain used in the present study [41–43]. Therefore, in line with conventional cognitive bias paradigms, these results could indeed indicate that mice displaying higher levels of stereotypy may have been in a more negative affective state [6,12,16], and perhaps in a more anxious state [6,12]. Further studies are needed however, to dissociate effects of affective state and behavioural inflexibility. This may be accomplished in future studies by also measuring recurrent perseveration separately.

When looking into the effects of the form of stereotypy performed on exploration of the maze, it became apparent that mice performing back-flipping were also the ones with the highest levels of stereotypic behaviour, thereby confounding the effects of form and level of stereotypy. Due to the low numbers of mice performing back-flipping (n = 3) and cage-top twirling (n = 4), we were unable to include stereotypy form in the statistical model. Therefore, we cannot exclude that the effects of stereotypy level on maze exploration reported above were strongly affected by the back-flipping mice. While the low sample sizes of some forms of stereotypies preclude firm conclusions, our results add to the existing literature, which suggests that different topologies of stereotypies may reflect different underlying mechanisms and may have different welfare implications for the animals [70,71]. Clearly, further studies are needed to examine whether different forms of stereotypies are associated with different patterns of exploration, and whether these differences are indicative of altered affective states.

Besides the possible welfare implications of stereotypic behaviour, our findings indicate that stereotypies may affect tests involving activity, exploration and responses to novelty and ambiguity, providing further evidence that stereotypy may affect research outcomes [72]. In fact, given that in mice both the form and expression of stereotypic behaviour vary among individuals, strains [42,43,69,73] and possibly laboratories, stereotypic behaviour could represent a major confound in research using laboratory mice.
